# Conopeptides from Cape Verde *Conus crotchii*

**DOI:** 10.3390/md11062203

**Published:** 2013-06-19

**Authors:** Jorge Neves, Alexandre Campos, Hugo Osório, Agostinho Antunes, Vitor Vasconcelos

**Affiliations:** 1 CIIMAR/CIMAR—Interdisciplinary Centre of Marine and Environmental Research, Rua dos Bragas 289, Porto 4050-123, Portugal; E-Mails: jorge.livramento@docente.unicv.edu.cv (J.N.); amoclclix@gmail.com (A.C.); aantunes@ciimar.up.pt (A.A.); 2 DECM—Department of Engineering and Sea Science, Cape Verde University, Mindelo CP 163, Cape Verde; 3 Institute of Molecular Pathology and Immunology of the University of Porto, IPATIMUP, Porto 4200-465, Portugal; E-Mail: hosorio@ipatimup.pt; 4 Faculty of Medicine, University of Porto, Porto 4200-319, Portugal; 5 Faculty of Sciences, University of Porto, Porto 4169-007, Portugal

**Keywords:** *Conus*, cone snails, conopeptide, conotoxin, Cape Verde

## Abstract

Marine Cone snails of the genus *Conus* contain complex peptide toxins in their venom. Living in tropical habitats, they usually use the powerful venom for self-defense and prey capture. Here, we study *Conus crotchii* venom duct using a peptide mass-matching approach. The *C. crotchii* was collected on the Cape Verde archipelago in the Boa Vista Island. The venom was analyzed using matrix-assisted laser desorption/ionization time-of-flight mass spectrometry (MALDI-TOF MS). About 488 molecular masses between 700 Da and 3000 Da were searched bymatching with known peptide sequences from UniProtKB protein sequence database. Through this method we were able to identify 12 conopeptides. For validation we considered the error between the experimental molecular mass (monoisotopic) and the calculated mass of less than 0.5 Da. All conopeptides detected belong to the A-, O1-, O2-, O3-, T- and D-superfamilies, which can block Ca^2+^ channels, inhibit K^+ ^channels and act on nicotinic acetylcholine receptors (nAChRs). Only a few of the detected peptides have a 100% UniProtKB database similarity, suggesting that several of them could be newly discovered marine drugs.

## 1. Introduction

Cone snails (genus *Conus*) are venomous predators belonging to the Conidae family. There are ~700 *Conus* species, all carrying complex arrays of peptide toxins in their venom [1]. *Conus* species normally live in tropical habitats of shallow water, on sand or near coral reefs and may cause lethal paralysis to their prey. Considered aggressive predators, cone snails are usually classified, depending on their prey, into three groups: vermivorous (worm-hunting), molluscivorous (other gastropods-hunting), and piscivorous cone snails (fish-hunting). However, some *Conus* can feed on hemichordates, bivalve mollusks and echiuroids, but a few species are considered generalist (e.g., *C. californicus*). *Conus* can be also dangerous to humans. The fish-hunting species *C. geographus*, have caused about three dozen fatalities in human poisoning cases [2,3]. The venom bioactive molecules, known as conopeptides or conotoxins (indiscriminate using of this work), are used to capture prey, as self defense from predators or to prevent competition [4,5]. Some authors have estimated that there are 100–200 distinct peptides per species, but recent work suggested that more than 1000 distinct conopeptides may be found per species [6]. These venoms mainly include linear peptides (usually disulfide-rich) and powerfully folded mini-proteins [7], exhibit various neuropharmacological properties with special incidence on ion channels and receptors [8,9]. Therefore, conopeptides have been considered powerful tools in neuroscience, and, for example, in December 2004, the synthetic version of the peptide ω-conotoxin MVIIA (commercial name Prialt^®^; Elan Pharmaceuticals, Inc., Dublin, Ireland) from *C. magus* has been approved by the United States Food and Drug Administration (FDA) to treat chronic pain in humans [10,11,12,13]. Despite this achievement, the overall knowledge of *Conus* venom proteins and peptides is scarce compared to other animal venom-producers (e.g., snakes, scorpions, spiders and sea anemones), thus providing a huge potential for the discovery of new pharmacological drugs [14]. The majority of *Conus* venom mini-proteins have a sequence length of 12–35 amino acids, normally with a high incidence of post-translational modifications. There are four classifications: (i) disulfide-rich conopeptides (conotoxins), which have two or more disulfides bridges, and disulfide-poor conopeptides, with one or none disulfide bond (conopressins, contryphans, conantokins, and contulakins); (ii) “gene superfamily” scheme that share a highly conserved sequence; (iii) “cysteine framework” scheme sorts them according to the arrangement of cysteines; and (iv) “pharmacological family” scheme reflects the target specificity of each conopeptide [2,9,10,15].

For the analysis of peptides the matrix-assisted laser desorption/ionization equipped with a time-of-flight (MALDI TOF/TOF) mass analyzer has been one of the most valuable analytical tools. This technique is relatively easy to perform (user friendly), is reliable and at the same time enables high-throughput sample analyses. Through MALDI-TOF, valuable data is generated [16], which contributes to the rapid discovery and characterization of new *Conus* marine drugs. The masses of peptides produced can be compared and “matched” to known sequences available on databases (e.g., UniProtKB and ConoServer). This methodology has been used to make available the identity of any protein whose full-length sequence is contained therein. Some peptides were identified within 0.5 Da (Daltons) of the predicted value, which was considered to be a sufficient criterion [17].

**Figure 1:**
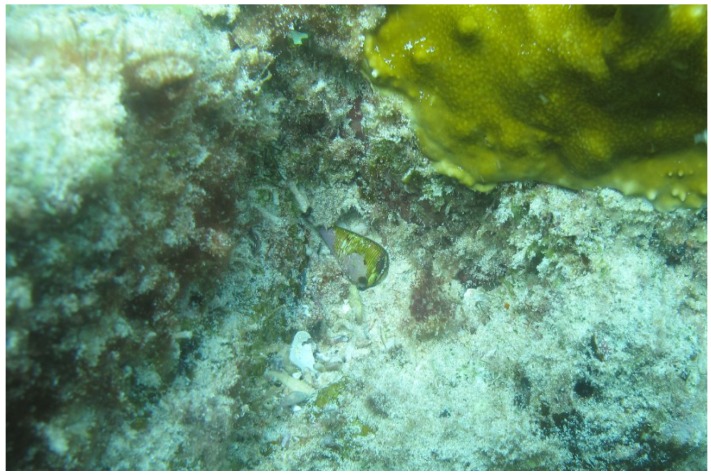
The endemic *C. crotchii* can be found only on Boa Vista island–Santa Mónica beach.

In the Cape Verde tropical Atlantic waters, there are 52 described *Conus* species, representing about 10% of the worldwide species diversity and only three are non-endemics (*C. ermineus*, *C. genuanus*, and *C. tabidus*). However, this high *Conus* diversity does not exist in other Macaronesian islands [18]. The conopeptides distribution by zoogeographic regions indicates a lack of information regarding the Atlantic Ocean. In this regard, 184 experimentally verified sequences have been reported from the Indo Pacific region and 25 from the Eastern Pacific region [19]. Only seven experimentally verified sequences from the Eastern Atlantic and Mediterranean regions, 22 from the Western Atlantic and Caribbean regions [19], and one conopeptide sequence from South African (with 18 endemic *Conus* species) have yet been reported [5]. In this study, we characterized the conopeptides from the *C. crotchii* venom duct using mass-matching approach (error ≤0.5 Da). *C. crotchii* have a heavy shell, with a greenish ground color, normally with fine spiral dark brown lines. It lives in shallow water (5 meters deep), and was observed only on south of Boa Vista island (Santa Mónica beach). The natural habitat is shown in Figure 1. Usually the *C. crotchii* length is nearly 30 mm. To our knowledge, this is the first description of conopeptides from the venom of a Cape Verde endemic *Conus*.

## 2. Results and Discussion

### 2.1. Peptide Mass Range Distribution

**Figure 2:**
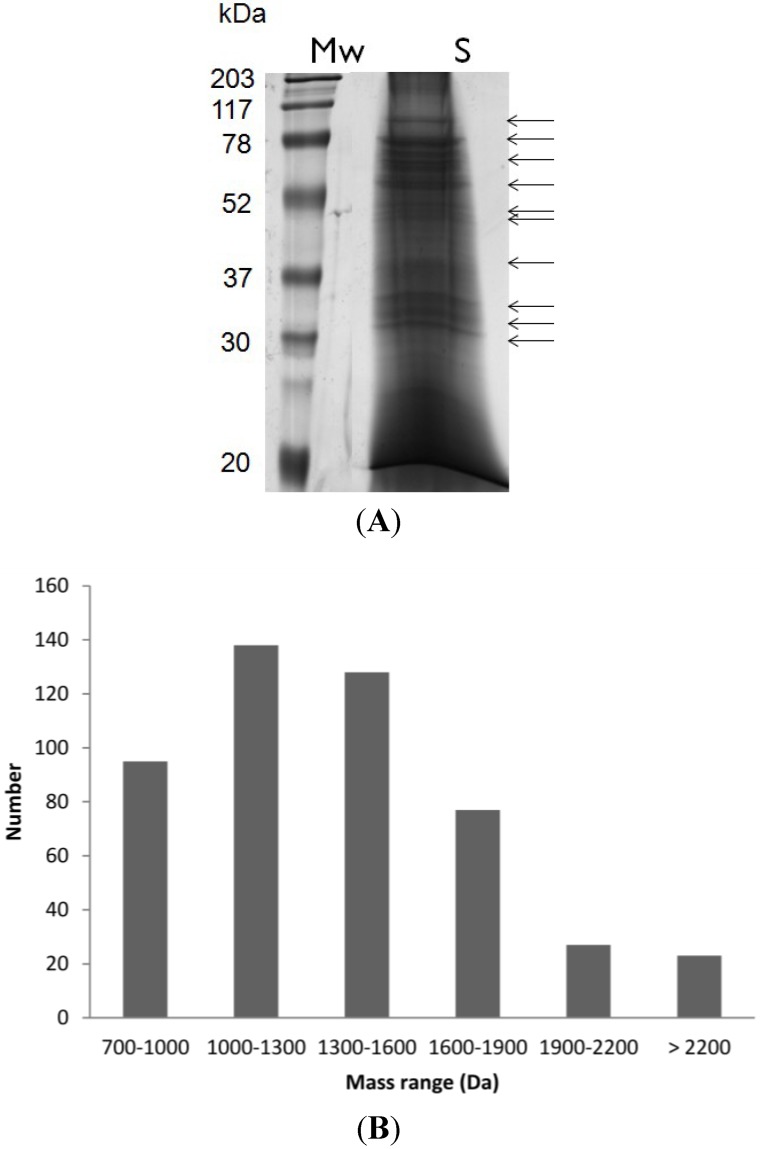
(**A**) Proteins separated by sodium dodecyl sulfate polyacrylamide gel electrophoresis (SDS-PAGE) from the venom duct of *Conus crotchii*. Mw: molecular weight, and S: sample; (**B**) Molecular mass range distribution of the 488 peptides detected by matrix-assisted laser desorption/ionization time-of-flight mass spectrometry (MALDI-TOF-MS) analysis.

The venom sample was first fractionated by SDS-PAGE (Figure 2A, SDS-PAGE only) and 2DE gel (data not shown). Each protein band was isolated and subjected to reduction, alkylation and trypsin digestion. Peptide samples were subsequently submitted to MALDI-TOF MS analysis, enabling the detection of 488 unique molecular masses, ranging from ~700 Da to ~3000 Da. The same technique was used to characterize *C. consors* peptides, rendering the detection of similar number of molecular masses (e.g., 550) [20]. The molecular mass distribution of the conopeptides in *C. crotchii* is shown in Figure 2B. Almost 90% of these masses were between 700 and 1900 Da and only 10% corresponded to large peptides. However, most of the peptides were between 1000 and 1600 Da. The *C. crotchii* molecular mass range is asymmetrically distributed, as described in the case of other cone snail venoms, namely from *C. consors* [20], *C. textile*, *C. imperialis* and *C. marmoreus* [6]. The mass range between 1000 and 2000 Da is the best range MALDI-TOF-MS detection for conopeptide with lower hydrophobicity [16].

### 2.2. Peptides Sequence

*Conus* venom peptides are classified into two groups: the disulfide-poor and the disulfide-rich [21]. The disulfide-rich peptides, also called conotoxins, contain one or more disulphide bridges. In this study, we are able to identify only disulfide-rich peptides by mass-matching (Table 1). These represented only 5% of all “*conomass*” detected. The 95% of conomasses not identified may thus represent an interesting pool of new conopeptides. Databases from National Center for Biotechnology Information (NCBI), UniProtKB, and Conoserver were used to match peptide masses predicted from sequences with a list of masses experimentally obtained in MASCOT search engine tool. This approach can facilitate the validation and accelerate the discovery of conopeptides [22] despite the high frequency and variability of post-translational modifications (PTM) displayed by conopeptides. Extensive PTM are common in *Conus* peptides like as hydroxylation of lysine to 5-hydroxylysine, cyclization of *N*-terminal glutamine to pyroglutamate or amidation of *C*-terminus [23]. Disulfide bonds are the most common PTM in conopeptides and are found in 220 of the 234 conopeptides isolated at the protein level [19].

**Table 1:** Peptide mass detected by MALDI-TOF-MS and putative sequence by mass-matching using MASCOT database research.

Conopeptide	Calculated Mass (Da)	Observed Mass (Da)	Sequence	Cysteine
1	1916.99 2042.78	1916.90 2042.72	TKTDDDVPLSSLRDNLK E**CC**EDGW**CC**TAAPLTGR	4
2	795.44 2232.96	795.48 2233.05	EQHLIR **CC**DFVKYVGMNPPADK**C**R	3
3	1701.89 2042.73	1701.80 2042.69	LWALMKGPRQCTPK DAP**C**DDNNQ**CC**SGLE**C**K	4
4	2545.05	2544.99	RPECCSDPRCNSTHPELCGGR	4
5	1076.56	1076.57	IRASEG**C**RK AVGLIDKMRR KGDR**C**GTHL**CC**PGLR	4
	1158.67 1786.82	1158.62 1786.88		
6	1790.81 1693.79	1790.87 1693.80	FQFLNF**CC**NEK ILEDIVSTALAT**CC**K	4
7	1687.88 1970.76	1687.88 1970.66	ASDGGNAAASDLIALTIK GCCSRPPCALSNPDYCG	4
8	798.32 1790.83	798.29 1790.89	CVGVCF G EQNKTCCGLTNGRPR	4
9	2042.76 798.34	2042.69 798.32	SGGACNSHDQCCINFCR KATSTCM	5
10	1169.51 1190.53	1169.55 1190.55	NFGDTRSCGR RGKPCPCCR	4
11	966.49 2205.99	966.46 2206.08	KCNRFNK IPNQKCFQHLDDCCSRK	4
12	985.45 1352.56	983.46 1352.59	RGHGRSCPG NGCTCVYHWR	3

The experimental MS spectra data was used in MASCOT database search tool to retrieve peptide sequences [24]. Here the molecular masses (monoisotopic) of the predicted peptides were compared with the estimated experimentally and the mass error was set as less than 0.1 Da. This high-quality conformity between predicted and observed masses lends confidence to the assignments (error ≤0.5 Da) [25]. To estimate the number of Cys residues in the peptide, disulfide bonds were reduced with dithiothreitol (DTT) and the Cys residues alkylated with iodoacetamide (IAA). This procedure is normally used for identifying disulfide-linked peptides and consequently the number of Cys using MALDI-TOF MS [26,27,28,29]. The results suggested the addition of carbamidomethyl group to each sulfur atom and a corresponding increase of 58 Da for each Cys in the peptide. The same chemical modification was suggested for peptide cal12a and cal12b from *C. californicus* [30]. 

### 2.3. BLAST Search for Conotoxins

**Figure 3:**
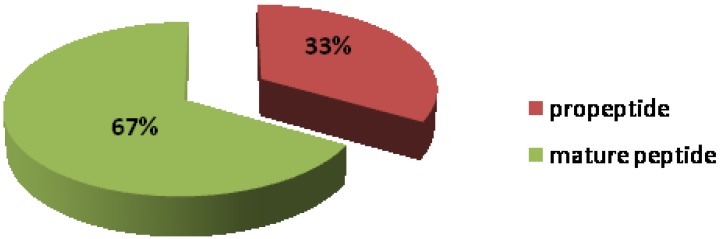
Distribution of peptide sequence identified as “Conoprotein”.

**Figure 4:**
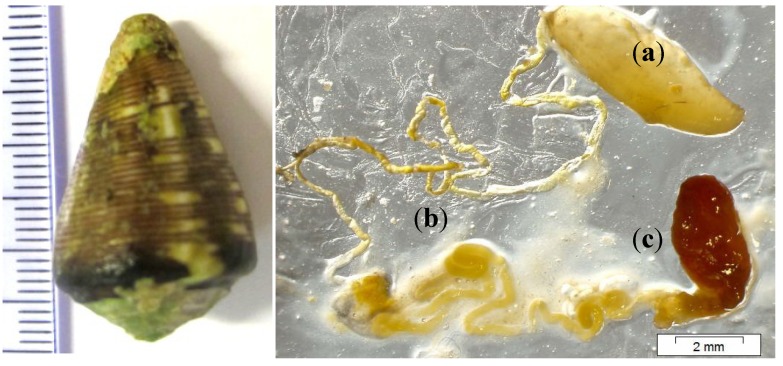
Shell and venom apparatus of *C. crotchii*: (**a**) Venom bulb; (**b**) Venom duct; (**c**) Venom gland.

All reduced and alkylated venom peptides from *C. crotchii* were analyzed by MS and amino acid sequence data were suggested by a MASCOT database search and blast analysis in UniProtKB. Peptides identified in the venom by MALDI-TOF MS are underlined (Table 2). Signal, propeptide and mature peptide regions are shown in red, blue and black, respectively (Table 2). For MALDI-TOF MS analysis, 33% and 67% propeptide and mature peptide were retrieved, respectively (Figure 3) from the venom duct and venom gland (Figure 4) that can likely be related with. The large percentage (67%) of peptide sequence corresponds to the mature peptide region due to the fact that the sample preparation was done with only the venom duct. Similarly, a total of 12 conotoxins were detected in the *C. crotchii* venom duct, considering only results with a protein 100% max. identity in UniProtKB (Table 2). A BLAST search on UniProtKB database resulted with an *E*-value between 1e-12 and 7e-30 and score bits between 151 and 257. The statistics from BLAST alignments was based on the marginally significant criterion *E*-value of 0.05, with normalized score of ~38 bits [31]. 

**Table 2:** Conotoxins identification by BLAST search on UniProtKB database; all 151 results have a 100% Max identity with the protein sequence. Signal, propeptide and mature peptide regions are shown in red, blue and black, respectively. Peptides identified in the venom by MALDI-TOF-MS—MASCOT database are underlined.

Conotoxin (Accession)	Protein sequence	*E*-Value ^a^
TxVA (P81755.2)	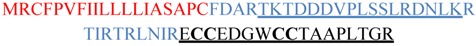	2e-12
im23.3 (D0PX86.1)	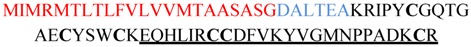	7e-21
Ec15a (B0KZ79.1)	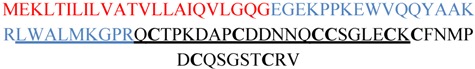	3e-28
Ai1.2 (P0CB08.1)	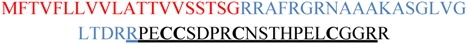	9e-17
Bu2 (P0CY61.1)	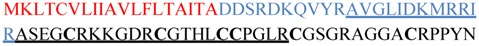	7e-30
Ca5.1 (P0C666.1)	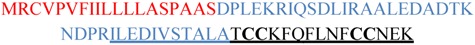	4e-22
PnMGMR-02 (Q9BP56.1)	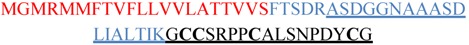	8e-30
VnMSGL-0123 (Q9BP59.1)	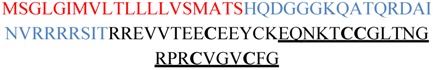	2e-15
Eb6.18 (C7T1P1.1)	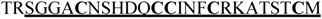	2e-19
Leo-O2 (P0C903.1)	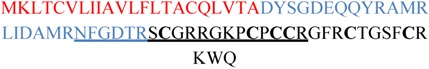	1e-12
PVIIA (P56633.2)	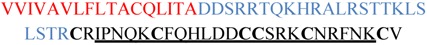	7e-20
VxXXB (P0C1W6.2)	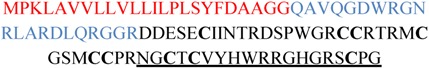	2e-14

^a^ The reported *E*-values were derived by the BLAST analysis.

All Cape Verde endemic *Conus* species are usually regarded as vermivorous, but it is not entirely clear that they are exclusively vermivorous [18]. The toxin’s sequence similarity results indicates that *C. crotchii* venom presents peptides from vermivorous (50%), but also molluscivorous (33%) and piscivorous (17%) *Conus* species. Among them, four conotoxins (Eb6.18, Leo-O2, Bu2, PVIIA) could be assigned to the O1-superfamily, three to the A-superfamily (im23.3, Ai1.2, PnMGMR-02), two to the T-superfamily (Ca5.1, TxVA) and one for each O2- (Ec15a), O3- (VnMSGL-0123) and D-superfamily (VxXXB), all of them previously described (Table 3). These results may suggest that the venom from *C. crotchii* has not only a dietary function but it is used for all kinds of environmental interaction as predators defense. On the other hand, it has already been demonstrated that size and diversity of the conopeptide gene superfamilies on vermivorous species differ significantly [5]. *Conus* peptides from the same superfamily share the typical arrangement of Cys residues in the mature toxin region, the “Cys pattern”. Each “Cys pattern” generally corresponds to a precise disulfide framework. However, within the same superfamily, thereissome altered loop spacing of amino acids between cysteines. For example, in the superfamily O, conopeptide μO-MrVIA (*C. marmoreus*) has interval residue number CX_6_CX_9_CCX_4_CX_4_C (6-9-4-4) and κ-PVIIA (*C. purpurascens*) has CX_6_CX_6_CCX_3_CX_5_C (6-6-3-5) [32]. Conopeptides from the superfamily O (cysteine framework “C-C-CC-C-C”) has a O1, O2 and O3 variation, can blockvoltage-gated Ca^2+^ channels and inhibits voltage-gated K^+^ channels [19]. However, the A-superfamily conopeptides (cysteine framework “CC-C-C”), one of the most studied superfamiles, together with the superfamilies O and T [19], can act on nicotinic acetylcholine receptors (nAChR) and can also block K^+^ channels [33]. 

**Table 3:** Conotoxins isolated from different *Conus* species.

Name	*C.* species	Diet	Superfamily	Family	Cys pattern (framework)	Reference
Ec15a	*C. emaciatus*	v	O2	unknown	C-C-CC-C-C-C-C (XV)	[31]
Ca5.1	*C. caracteristicus*	v	T	unknown	CC-CC (V)	[34]
im23.3	*C. imperialis*	v	A	unknown	C-C-C-CC-C (XXIII)	[35]
Eb6.18	*C. ebraeus*	v	O1	unknown	C-C-CC-C-C (VI/VII)	[36]
Leo-O2	*C. leopardus*	v	O1	unknown	C-C-CC-C-C (VI/VII)	[37]
VxXXB	*C. vexillum*	v	D	α	C-CC-C-CC-C-C-C (XX)	[38]
TxVA	*C. textile*	m	T	є	CC-CC (V)	[39]
Ai1.2	*C. ammairalis*	m	A	α	CC-C-C (I)	[31]
PnMGMR-02	*C. pennaceus*	m	A	α	CC-C-C (I)	[31]
VnMSGL-0123	*C. ventricosus*	m	O3	unknown	C-C-CC-C-C (VI/VII)	[31]
Bu2	*C. bullatus*	p	O1	unknown	C-C-CC-C-C (VI/VII)	[40]
PVIIA	*C. purpurascens*	p	O1	κ	C-C-CC-C-C (VI/VII)	[41]

v: vermivorous; m: molluscivorous; p: piscivorous.

## 3. Experimental Section

### 3.1. Cone Snail Specimen and Venom Extraction

The *Conus crotchii* were obtained from the Boa Vista Island in the Cape Verde archipelago. The venom ducts were dissected on ice and the venom duct removed and diluted in 500 μL of 0.1% formic acidand stored at −80 °C until use. The image was obtained (Figure 4) using magnifying glass Olympus DP72, DF lenses mode (Tokyo, Japan). The venom ducts were mechanically disrupted by ceramic beads (diameter 1.4 mm) using Precellys 24 homogenizer (5400 rpm, 2 × 15 s; Bertin, Montigny-le-Bretonneux, France). Ceramic beads and insoluble materials were removed by centrifugation at 4 °C (16× *g* for 10 min, twice). After centrifugation, all venom extract were immediately stored at −20 °C prior to analysis.

### 3.2. Sample Fractionation, SDS-PAGE

Protein samples were mixed with loading buffer with Tris-HCl (0.25 M) pH 6.8, SDS (6%, w/v), glycerol (30%, v/v), β-mercaptoethanol (6.25%, v/v) and proteins separated by sodium dodecyl sulfate polyacrylamide gel electrophoresis (SDS-PAGE) in homogeneous gels (12% acrylamide) according to Laemmli [42]. Gels were stained with colloidal Coomassie Blue [43], gel images acquired using GS-800 calibrated scanner (Bio-Rad, Hercules, CA, USA) and the protein profiles analyzed using the Quantity One software (Bio-Rad, Hercules, CA, USA).

### 3.3. Protein Reduction, Alkylation and Trypsin Digestion

Proteins were isolated from the SDS-PAGE gels and submitted to *in-gel* reduction and alkylation to disrupt disulfide bonds [44,45], and thereafter to trypsin digestion. Reduction was achieved by adding 50 μL of DTT (10 mM) prepared in NH_4_HCO_3_ (100 mM, pH 8.0) to the protein samples followed by incubation during 45 min at 56 °C. For alkylation the dithiothreitol (DTT) was replaced by 50 μL IAA (55 mM) prepared in NH_4_HCO_3_ (100 mM) and the protein samples incubated during 30 min in the dark. For trypsin digestion dried protein gel bands were incubated with 6.7 ng trypsin/μL during 30 min in ice. Thereafter, the excess of trypsin solution was removed and 5–25 μL of NH_4_HCO_3_ (50 mM) was added in order to cover the gel pieces. Trypsin digestion proceeded overnight (15 h) at 37 °C. The solution (supernatant) containing the peptides was subsequently collected into an eppendorf tube and stored at −20 °C. 

### 3.4. MALDI-TOF-MS Analysis

The peptide samples were concentrated and cleaned according to the manufacturer’s instructions on a micro C18 ZipTiP column (Millipore, Bedford, MA, USA). The peptides were eluted directly onto the MALDI plate using the matrix α-cyano-4-hydroxycinnamic acid (α-CHCA) at 5 mg/mL prepared in ACN (50%), and formic acid (0.1%). Peptide mass spectrometry analyses were performed by MALDI-TOF/TOF (4700 Proteomics Analyzer, AB SCIEX, Foster City, CA, USA) method described [46,47,48] in reflector positive mode (700–4000 Da). The experimental mass spectra were searched against the UniprotKB protein sequence database with the Mascot (Matrix-Science, London, UK) algorithm, integrated in the GPS Explorer software (AB SCIEX, Foster City, CA, USA). The search parameters were up to two maximum trypsin missed cleavages, mass tolerance of 50 ppm, cysteine carbamidomethylation (fixed modification), methionine oxidation (variable modification) and a charge state of +1. 

## 4. Conclusions

In this work we characterized the peptide profile from *C. crotchii* using MALDI-TOF and mass-matching. The number of molecular masses studied here resembles the outputs from other studies performed in the genera *Conus* enabling us to validate our approach. We were able to identify several disulfide-rich conotoxins in *C. crotchii* venom duct samples that belong to O1-superfamily (Eb6.18, Leo-O2, Bu2, PVIIA), A-superfamily (im23.3, Ai1.2, PnMGMR-02), T-superfamily (Ca5.1, TxVA) and O2- (Ec15a), O3- (VnMSGL-0123) and D-superfamilies (VxXXB). Taking into consideration the mode of action of some of these conotoxins we may consider that the high diversity of conotoxins may not only be related to diet but with all kinds of environmental interaction as predators’ defense. These putative conotoxins may block voltage-gated Ca^2+^ channels, inhibit voltage-gated K^+ ^channels and act on nAChRs [15,30]. A large number of masses were not assigned in this work. This promises new research potential and the discovery of new bioactive molecules from Cape Verde *Conus* species.
